# A reservoir induced earthquake swarm in the Central Highlands of Sri Lanka

**DOI:** 10.1038/s41598-022-22791-z

**Published:** 2022-10-29

**Authors:** Pasan Herath, Januka Attanayake, Kalpna Gahalaut

**Affiliations:** 1 Institute of Geophysics, Victoria University of Wellington, Wellington, New Zealand; 2 School of Geography, Earth and Atmospheric Sciences, University of Melbourne, Melbourne, Australia; 3 CSIR-National Geophysical Research Institute, Hyderabad, Telangana India; 4 Present Address: Department of Earth and Environmental Sciences, University of Ottawa, Ottawa, Canada

**Keywords:** Tectonics, Seismology

## Abstract

An anomalous seismic sequence of five small (M_W_ < 3) felt earthquakes occurred between 29 August 2020 and 05 December 2020 around the Victoria Reservoir in the central highlands of Sri Lanka that clearly exceeded the established national background seismic rate. Using seismic waveform template-matching and a newly developed single-station earthquake location method based on travel-time back-projection, we detected an additional co-located 23 microseismic events, of which 18 occurred within the same period as the felt events. This hitherto undetected seismic swarm defines a seismogenic zone beneath the western flank of the reservoir between 1.5 and 3 km depths. The reservoir-induced peak stresses, resolved on E-W striking faults, predicted from the poroelastic theory that include both drained and undrained crustal responses are ~ 15 kPa in an area overlapping the seismogenic zone, which, together with the physical and spatio-temporal characteristics of the seismic swarm, establish a causal link between reservoir-induced stresses and the earthquake swarm with implications to seismic hazard. This is the first record of induced seismicity in Sri Lanka. The newly developed efficient computational workflows with minimal operational costs described in our study provide a blueprint for monitoring reservoir-induced seismicity in developing countries with severe resource limitations.

## Introduction

Determining the onset, mechanisms, and seismic hazard risk of reservoir-triggered seismicity (RTS) is difficult. However, the human-induced earthquake (HiQuake) database reports that about 15% of the 1235 globally distributed projects that it contains have produced RTS^1,2^. RTS is frequently observed at the initial impoundment stage and manifests as elevated seismic rates in the shallow crust that decays relatively rapidly (months-to-few years) to pre-impoundment background rates with a straightforward causal relationship between crustal poroelastic response and perturbations to the ambient stress field from instantaneous loading and pore pressure diffusion^3^. In some other instances, delayed pulsating episodes of RTS associated with crustal response to periodic reservoir water level fluctuations have been identified^4–7^. In still other instances, RTS is protracted for several years to decades^8^ and occasionally leads to deeper, larger triggered events. Earthquakes that have occurred proximal to Koyna, India (1967 M6.3), Kremasta, Greece (1965 M6.2), Xingfengjiang, China (1962 M6.1), Kariba, Zambia-Zimbabwe (1963 M6.1), Aswan Lake (1981 M5.3), and Thomson Dam (1996 M5), Australia are classic examples of this latter case^9–12^.

**Figure 1:**
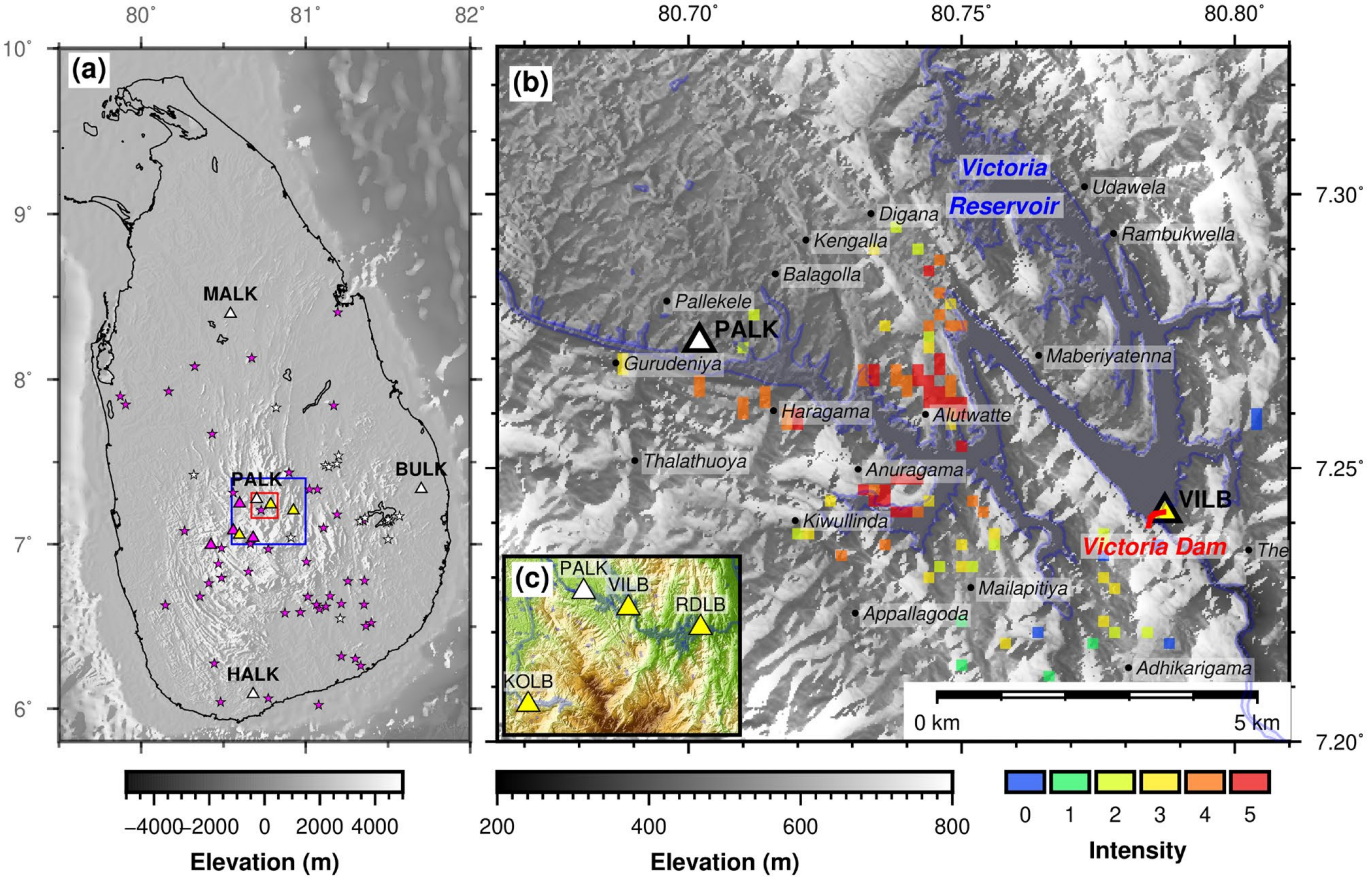
Historical seismicity in Sri Lanka and felt intensity map around the Victoria Reservoir for the 29 August 2020 earthquake. (**a**) Historical seismicity of Sri Lanka. Purple stars are earthquake epicenters from a temporary network (purple triangles) that operated between 1983 and 1984^16^. White stars are earthquake epicenters from the modern seismograph network (white triangles). Red rectangle indicates the extent of (**b**). The blue rectangle indicates the extent of (**c**). (**b**) Felt intensity on an arbitrary categorical scale of the earthquake that occurred on 29 August 2020 in close proximity to the Victoria Reservoir^52^. (**c**) Locations of the broadband seismograph (PALK) and accelerometers at dam sites in the central highlands (VILB-Victoria left bank), RDLB (Randenigala left bank), KOLB (Kotmale left bank). Figures made with Generic Mapping Tools^61^.

Following the occurrence of a rare series of felt, yet small earthquakes (M_W_ < 3, determined in this study) proximal to the Victoria Reservoir in the central highlands of Sri Lanka between August and December of 2020 (Fig. 1), questions were raised about the origin of this seismic sequence because it exceeded established national background seismic rates. Testing the RTS hypothesis, as a measure of ensuring the safety of infrastructure at the Victoria Reservoir and the community around it, is critical given the prominent role the reservoir plays in the national and local economies. The Victoria Reservoir is a major component of the multi-purpose accelerated Mahaweli Master Plan^13^, which envisaged the construction of a series of reservoirs along the 335 km long Mahaweli Ganga, the longest and largest river in Sri Lanka that has a catchment area nearly equivalent to 1/6 of the area of the country^14^, to develop and irrigate 3650 km^2^ of agricultural land and generate 600 MW of hydroelectricity. The Victoria Reservoir, alone, is designed to develop and irrigate a land area of 212 km^2^ and generate 210 MW of hydroelectricity^15^. The double curvature Victoria Dam (Fig. 1b) was a state-of-the-art structure at the time of its completion in 1984 and has a crest length of 525 m and a maximum height of 122 m above the foundation^15^. The reservoir has a total catchment area of 1869 km^2^ with a gross capacity of 728 Mm^3^ and an active storage capacity of 698 Mm^3^ that can generate 780 GWh per year^13^.

The five felt earthquakes that occurred in the vicinity of the Victoria Reservoir are anomalous considering Sri Lanka’s background seismic rate. Existing sparse Island-wide event catalogues indicate that about 30 microearthquakes (M ≤ 2.5) occur every year and 1–2 earthquakes, usually with magnitudes less than 3, are felt on average^16^. The largest known earthquake in Sri Lanka with a magnitude of 6+ in 1615 is interpreted from historical damage information and assumed to have occurred near Colombo^17^. Since 1615, only three other events have been reported with a magnitude ≥ 5 in the country although numerous events with M ≥ 5 have occurred in the near-offshore environment in that period. The low seismic rate reported in and around Sri Lanka is thus consistent with the expectations in a stable continental region. While this sparse seismic catalogue has been used to model the probabilistic seismic hazard of Sri Lanka^18^, the implications of the presence of neotectonic (≤ 8 Ma) and older faults^19^ that are apparently not associated with contemporary seismicity have not been considered to form a more realistic perception of hazard. One such implication is that they appear, solely based on fault lengths, to be able to produce M ≥ 5 earthquakes if their entire fault lengths were to rupture in single events. Another concern more relevant to this study is whether they can promote RTS as long-term stable crustal stresses are perturbed by the impoundment of reservoirs or cyclical reservoir water level changes along the Mahaweli Ganga.

The primary requirement for establishing such a causal link between seismicity and reservoir-induced stresses is developing precise earthquake catalogues, for which high-performing seismic monitoring solutions are needed. Ideally, a seismometer network with the nearest event-station distance equivalent to 1–2 times the focal depth of events and an azimuthal coverage preferably with gap angles less than 120˚ and about 8-10 high-quality P- and S-wave travel-time measurements are required to precisely invert for earthquake locations and characterise sources^20–23^. Together with accurate subsurface seismic velocity models, these criteria ensure a level of resolution of source parameters needed to conduct RTS analysis.

However, these ideal seismic monitoring conditions rarely exist around reservoirs unless RTS is identified as a potential risk early on at the design stage. In particular, seismic monitoring for RTS in developing countries is quite challenging due to inadequate financial investments and a lack of physical and human resources. Such limitations render much-needed seismic monitoring suboptimal or non-existent^24^ despite rapid urbanisation undertaken in these countries that involves the construction of large-scale critical infrastructure such as dams and reservoirs. Foregoing seismic monitoring due to a lack of resources inevitably leaves the communities around such critical infrastructure exposed to the hazard of RTS. To overcome these challenges disproportionately affecting developing countries, new efficient monitoring methods that can optimally exploit available limited resources need to be explored.

Conditions for monitoring seismicity of the area encompassing the Victoria Reservoir are similar to those that exist in other resource-scarce developing countries. There is a heavy reliance on one broadband seismometer (PALK) located in a 90 m deep borehole, which is part of a four-element national broadband array (Fig. 1a), to monitor local seismicity in the vicinity of the Victoria Dam (Fig. 1b). The accelerometers fitted to Victoria Dam can be useful for monitoring seismicity but their performance is not well monitored nor is its continuous data made available through an open-access license. These conditions preclude meaningful reservoir-scale seismic monitoring with conventional event detection and location methods, thereby preventing investigations of RTS. To overcome these limitations, we have designed a new single-station method, in which seismic events are detected with template matching and located using a precise travel-time back-projection technique. Deploying this new method, we assembled a new high-precision seismic catalogue for the area of interest, based on which seismicity is analyzed. We then use predictions from poroelastic theory to test whether there is a causal link between this anomalous seismic sequence and reservoir-induced stresses.

## Results

### Seismic swarm

Using our template-matching technique (see “Methods”), we detected a total of 23 new events in addition to the five felt events (Supplementary Table S1). While the felt events occurred between 29 August 2020 and 05 December 2020, we detected events as early as 14 February 2020 and as late as 26 May 2021. We note, however, that the most intense episode of this seismic sequence is highly localised in space and time with 23 out of the 28 earthquakes in our event catalogue occurring within the time window during which the felt earthquakes were reported. The events that do not fall within this time window are likely associated with the background seismic rate. The fact that no events were detected between 01 January 2012 and 13 February 2020 in the continuous data stream of PALK with matching event templates lends confidence to our claim that this seismic sequence is indeed a unique and anomalous episode of seismic activity deviating from background seismicity, where the highly spatially localised seismic rate between 29 August and 05 December 2020 exceeds the established island-wide seismic background rate of about 30 M ≤ 2.5 earthquakes a year with 1-2 felt events^16^. Likely, we have not detected all events in the swarm primarily because we adopted a higher waveform similarity cut-off of 0.8 to ensure the reliability of detections.

**Figure 2:**
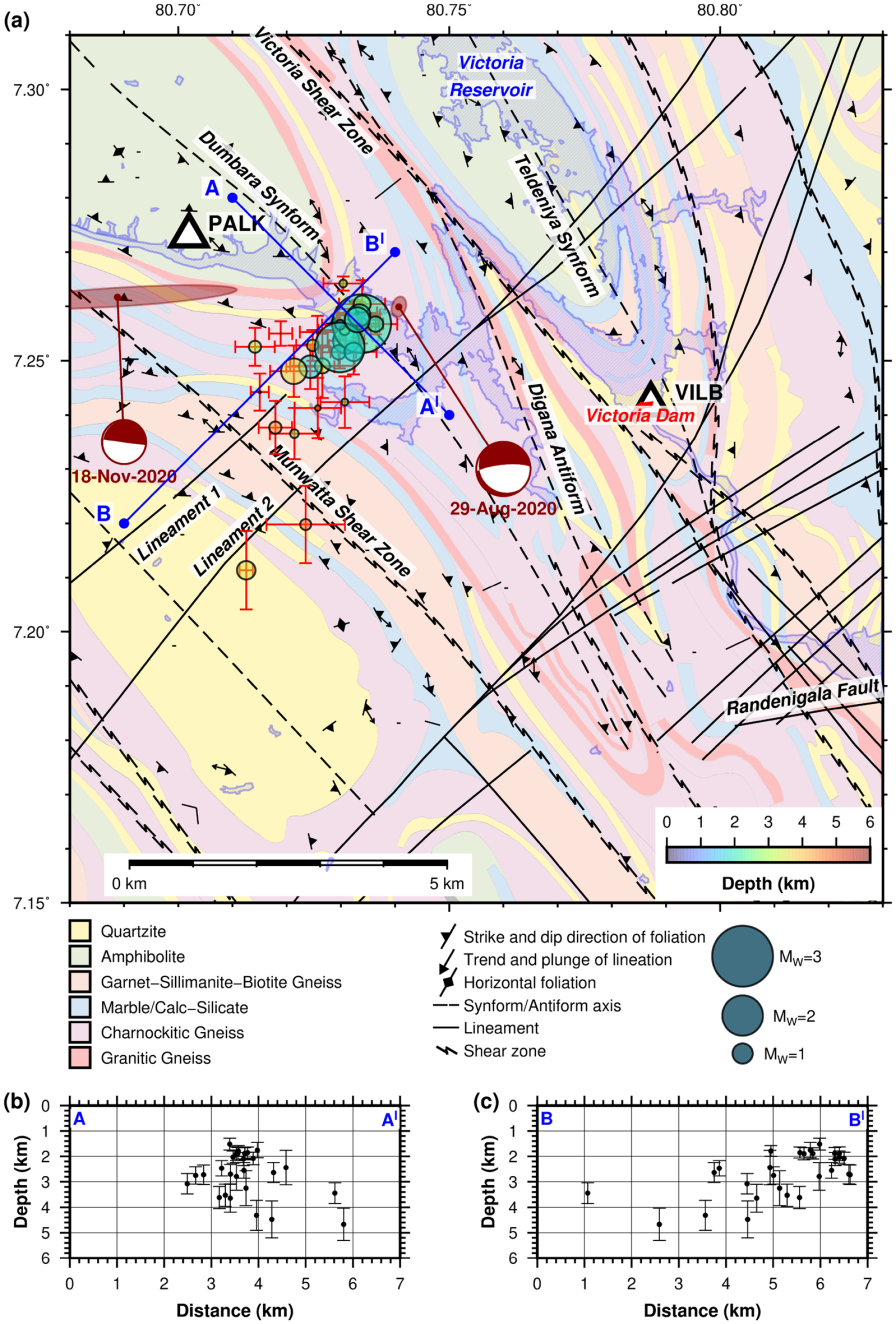
Centroids of the seismic swarm and the geology and structure of the area. (**a**) Epicentres of earthquakes. Error bars (red) indicate the standard deviation in the epicentre estimates from the single station method. Red ellipses indicate the error ellipses of the epicentres of the two earthquakes on 29 August and 18 November 2020 from travel-time inversion. (**b**) Profile along AA^I^. (**c**) Profile along BB^I^. Error bars in (**b**) and (**c**) indicate standard deviation in depth estimates from the single-station method. Figure compiled using Generic Mapping Tools^61^.

The precise locations estimated using travel-time back-projection confirm that this anomalous earthquake sequence is confined to an area of radius 2-3 km on the western flank of the Victoria Reservoir at depths of about 1.5 km to 4.7 km (Figs. 1b, 2). The temporal distribution of moment magnitudes (M_W_) (Supplementary Table S1 and Fig. S1), where smaller events precede the largest event, is consistent with the characterisation of this seismic sequence as a swarm rather than a mainshock-aftershock sequence^25^. The difference in the event magnitudes between the two largest earthquakes of 0.5 (29 August 2020; M_W_ 2.9 and 18 November 2020; M_W_ 2.4) is also consistent with our characterisation of the series of earthquakes as a seismic swarm because the largest aftershocks are smaller by about one magnitude unit than the mainshock in a typical mainshock-aftershock sequence^26^. The identification of this seismic episode as a swarm rather than a mainshock-aftershock sequence is important because the former is generally associated with RTS^3,27^, whereas the latter is typical of natural release of tectonic stress. Our ability to detect events as small as M_W_ -1.3 is a testament to the utility of template matching method in a resource-scarce environment and the quality of data recorded by PALK in a 90 m deep borehole. In the absence of a country-specific operational ground motion attenuation equation, we computed event magnitudes from a ground motion prediction equation^18^ applicable to seismic hazard analysis derived from a relatively sparse dataset, for which reason we believe that the event magnitudes might have uncertainties of several tenths of a decimal point.

### Reservoir-induced stress changes and fault stability

**Figure 3:**
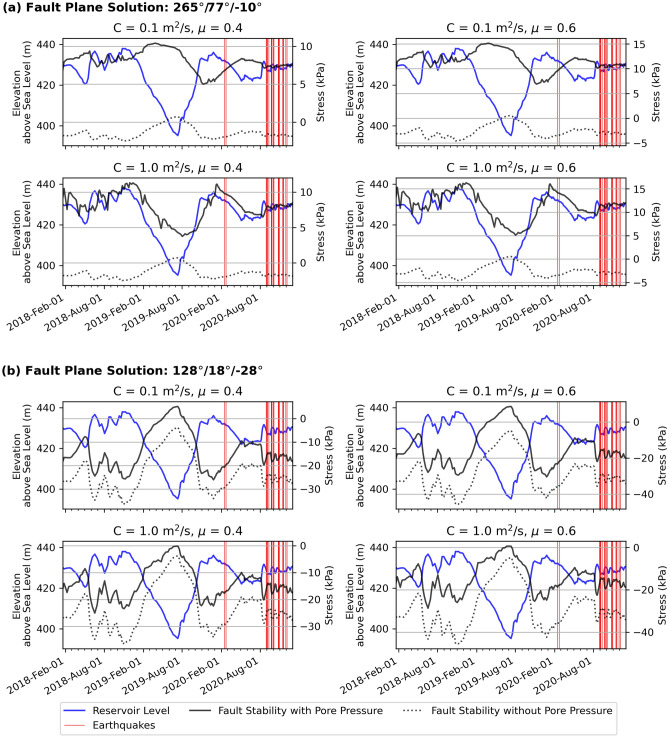
Temporal variation of change of fault stability. (**a**,**b**) The change of fault stability predictions for fault plane solutions of the largest earthquake on 29 August 2020 in dry (without pore pressure; dotted line) and wet (with pore pressure; black solid line) conditions. Failure is encouraged if the fault stability is positive. The solutions are for longitude = 80.73842° and latitude = 7.26497° (epicenter of the 29 August 2020 M_W_ 2.9 event) and 2 km depth. Figure made with Python Matplotlib^62^.

In Fig. 3, we show the evolution of change of fault stability for fault plane solutions of the largest earthquake (29 August 2020) (see “Methods”) with and without pore pressure computed using hydraulic diffusivity (C) and fault friction (μ) values appropriate for intraplate settings. By definition, shear failure of a fault is promoted if the change of fault stability is positive and it is impeded when fault stability is negative.

For the steep fault striking 265° with an oblique normal sense of slip (Supplementary Table S2 and Fig. 2a), we find that the change of fault stability is negative when only instantaneous elastic stresses are considered, whereas it is positive when pore pressure is accounted for. Importantly, the induced stresses (black solid line) peak at ~15 kPa with pore pressure around the time when the reservoir water level (blue solid line) fluctuates significantly. The exact time at which induced stresses peak depends on C, and not on μ, where peak stresses approximately coincide with the lowest reservoir water levels for C = 0.1 m^2^/s (Fig. 3a). On the other hand, if C = 1 m^2^/s, peak induced stresses approximately coincide with the onset and termination of the major water level fluctuation. In either case, the positivity of fault stability with peak induced stresses of ~15 kPa is a decisive prediction because it suggests that these faults were brought to failure as the Coulomb failure threshold (~ 10 kPa)^28,29^ has been exceeded. To the contrary, the change of fault stability of the shallow-dipping fault striking 128˚ (Supplementary Table S2) with a sense of slip similar to the E–W striking faults remains negative at all times except for brief periods when peak induced stresses reach < 5 kPa for all variables considered here (Fig. 3b), implying that faults striking NNW-SSE were not activated due to reservoir water level changes.

We also computed the change of fault stability for the fault plane solutions of the 18 November 2020 earthquake, which has an oblique reverse sense of slip (Fig. 2a, Supplementary Table S2 and Fig. S2). Again, we find that the stability of the steep fault striking E-W is positive for significant lengths of time and, with the action of pore pressure, induced stresses reach 5–10 kPa, whereas SSW–NNE striking fault has a negative change of fault stability (Supplementary Fig. S2). Combining the results of the 29 August 2020 and 18 November 2020 events, we find that steep faults striking E–W are preferentially brought to failure by the action of pore pressure. This poroelastic theoretical prediction establishes a causal link between the detected earthquakes and a major reservoir water level fluctuation if the E–W striking fault plane solutions are representative of fault geometries of the entire seismic swarm.

**Figure 4:**
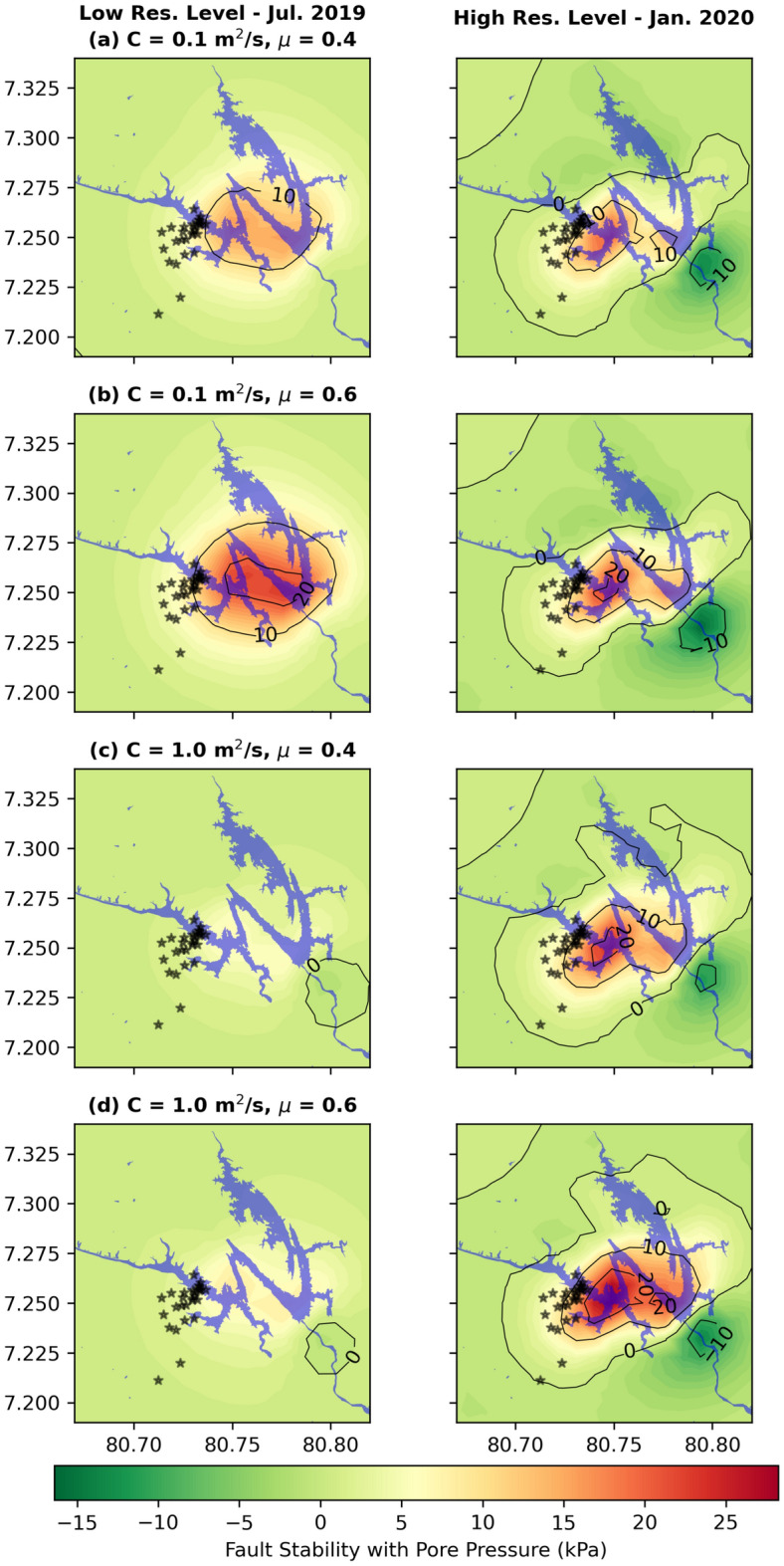
Spatial variation of change in fault stability. (**a**–**d**) Spatial stability of the fault plane striking 265° of the largest earthquake on 29 August 2020 earthquake with pore-pressure for diffusivities (C) of 0.1, 1.0 m^2^/s and coefficients of friction (μ) of 0.4, 0.6 at the low reservoir level (left column) in July 2019 and high reservoir level (right column) in January 2020 at a depth of 2 km (see Fig. 3 for reservoir level time series). Note the increase in fault stability when the reservoir level is high in January 2020. Black stars are earthquake epicentres in Supplementary Table S1. Blue coloured region is the Victoria Reservoir. Figure made with Python Matplotlib^62^.

As previously pointed out, we observe that peak induced stresses are observed around a major water level change in the reservoir (Fig. 3, Supplementary Fig. S2). It peaked in November 2018 (438 m), following which dropped to a minimum of 395 m in July 2019. The next peak of 436 m was reached towards the end of December 2019, completing a full cycle (Fig. 3, Supplementary Fig. S2). Because reservoir water level data prior to January 2018 is not available to us, we cannot confirm whether the observed cycle of water level change is typical for the Victoria Reservoir. If it is, our predictions suggest that a water level fluctuation of 40 m can induce substantial subsurface Coulomb stress variations of 10–15 kPa. To demonstrate this, we plotted reservoir-wide lateral variations of induced stress assuming a fault orientation similar to the E–W fault plane solution of the 29 August 2020 earthquake for July 2019 (minimum water level) and January 2020 (a peak water level) (Fig. 4). It confirms that Coulomb failure thresholds are consistently met in the swarm region on the western flank of the Victoria Reservoir.

## Discussion

The source parameters and spatio-temporal characteristics of the seismic swarm, together with our poroelastic predictions of induced stress, establish a causal link between the major water level fluctuation of ~ 40 m and the detected seismic activity proximal to the Victoria Reservoir assuming that E–W striking steep faults have failed. In this discussion, we focus on three key aspects of RTS around the Victoria Reservoir: (1) characteristics of the seismogenic zone; (2) timing of the onset of seismic activity; and (3) implications to seismic risk.

(1) Characteristics of the seismogenic zone: Epicentres of all 28 earthquakes estimated from the single-station travel-time back-projection method define a tightly bound seismogenic zone beneath the western flank of the Victoria Reservoir (Fig. 2a). Estimated hypocentral depths define the vertical extent of this seismogenic zone from ~ 1.5 to ~ 4.7 km with a majority of events clustered between ~1.5 and 3 km (Fig. 2b,c). These two features of the seismogenic zone, i.e. its tightly bound location at the periphery of the reservoir and shallow depth, are consistent with previous observations of typical RTS^8,27^.

Probabilistic focal mechanism solutions together with regional bedrock structural maps provide further insights into the geometry of the geologic structures that may have activated within the newly detected seismogenic zone. Our fault plane solutions are consistent with the geometry of the dominant deformational structures in the region (Fig. 2a). The location of the seismic swarm intersects the axis of the Dumbara Synform which is the main deformational structure aligned in a NNW-SSE direction. This structure is bound by Munwatte and Victoria Shear Zones and on the southwest and northeast respectively. On the other hand, the main E-W oriented deformational feature in the region is the neotectonic steeply-dipping Randenigala Fault^19^ located at about 12 km southeast of the seismic swarm (Fig. 2a), which establishes a structural geologic condition that favours the formation of steeply-dipping E–W striking faults under the neotectonic (< 8 Ma) stress field characterised by N–S compression. We also note that bedrock foliations in the study area defined by strong compositional layering with alternating mafic and phyllosilicate-rich bands of minerals^30^ are also well aligned with the E–W striking fault plane solutions at the epicenters of the two largest earthquakes. The presence of these two structural features (i.e. steeply-dipping E–W striking faults and subparallel foliations) can explain the occurrence of earthquakes under reservoir-induced stresses as predicted in our poroelastic computations. It is possible that mechanically weaker phyllosilicate-rich bedrock foliations in the southeastern limb of the Dumbara Synform (Fig. 2a) control shallow earthquake rupture pathways as seen elsewhere in intraplate environments^31^. We also highlight that a majority of earthquakes are occurring where the Dumbara Synform axis and Lineament 1 are intersecting each other (Fig. 2a). Such fault intersection points have been identified as locations where stresses accumulate^32^, possibly further weakening favourably oriented faults.

Our change of fault stability computations confirm that pore pressure plays a decisive role in driving induced stresses beyond Coulomb failure thresholds. We computed critical Coulomb stresses on the faults based on an induced seismicity source model^33^ applicable to geothermal reservoirs to be between 1.8 MPa and 34 MPa for our earthquake dataset assuming a shear modulus of 25 GPa and a critical strain rate of 6 × 10^−7^ s^−1^, which are two to three orders of magnitude greater than the reservoir induced stress. This implies that the crust within the seismogenic zone is in a critically stressed state and that only a small stress perturbation such as that produced by the water level fluctuation of the Victoria Reservoir is needed to trigger seismic failure^34^.

(2) Timing of Reservoir-Induced Seismicity: The timing of RTS depends on *in situ* heterogeneous tectonic stresses, the evolution of coupled poroelastic response of the crust (i.e. undrained and drained response), and the critical frictional strength profile of pre-existing faults^4,35–38^. Different interaction pathways of these processes determine how and when shear failure of pre-existing faults is activated, analysis of which enables identifying dominant driving mechanisms of RTS^38–40^. Despite a majority of RTS episodes have generated less harmful small M < 4 earthquakes^1,2^, determining these driving mechanisms help avoid or manage possible significant seismic hazard scenarios, particularly when reservoir-induced stress perturbations are anticipated to intersect known active or dormant deformational structures^32,41^.

The timing of the seismic swarm investigated here is intriguing and we believe that it is intimately related to the action of pore water based on the decisive role it plays in elevating induced stresses beyond the Coulomb failure threshold. Pore water is described to have a two-fold impact on faults. On the one hand, the action of pore water pressure can produce a mechanical effect on pre-existing faults, whereby frictional strength of faults is weakened due to a reduction in effective normal stress^42^. On the other hand, pore water can degenerate pre-existing faults through chemical interactions. While spatio-temporal distribution of RTS is determined by the mechanical effects of pore water, the onset of seismicity is influenced by rock-water chemical interactions^43^.

This understanding of how pore water influences faults is important to interpret the timing of RTS associated with the Victoria Reservoir. To our knowledge, there is no previous record of induced seismicity proximal to the Victoria Reservoir including the 1983–1984 period during which the first filling was completed. Our study suggests that the detected seismic swarm is indeed unique as no event has been detected from January 2012 through February 2020 prior to the emergence of this swarm. This raises a question about the timing of RTS, as the present seismic swarm has occurred nearly 36 years after initial impoundment. We propose two hypotheses to explain this delayed response of the crust to reservoir induced stress:

Hypothesis 1—While we are confident that felt events with a frequency similar to that reported here have not occurred in the last 36 years, previous (i.e. prior to 2012) RTS episodes may have gone undetected if imperceptible induced microseismic events have occurred during that time because no systematic monitoring was in place. Note that detecting some of the smallest events in our catalogue was only possible with our template-matching technique, which is the first instance that it was used in Sri Lanka. Such detections were also partly facilitated by significantly reduced ambient noise conditions present in the 90 m deep borehole in which PALK is installed. If this hypothesis is correct, it may mean that RTS is triggered by major water level variations of the Victoria Reservoir.

Hypothesis 2—The present seismic swarm is the first RTS sequence associated with the Victoria Reservoir. This suggests that pre-existing deformational structures remained unperturbed up until 2020, consistent with the observation of slow strain rates and strong crustal strength in intraplate regions^44,45^. It may then be a case of continuous chemical interactions between rock and pore water weakening these structures^43^ since the filling of the Victoria Reservoir to a point where mechanical action of pore water could overcome frictional strength of faults. In either case, we reiterate that pore water pressure plays a significant role.

The time lag of about 10 months between peak induced stress (~ 15 kPa) and the onset of seismicity may be explained by hydraulic diffusivity (C) that controls the speed at which pore pressure diffusion front expands. Similar time lags between peak stress perturbations and the onset of RTS have been reported around the Koyna fault zone^7^. We also note a rapid positive change in fault stability for C = 1.0 m^2^/s towards the end of August 2020 corresponding to a rapid increase in the reservoir water level (Fig. 3a). If this rapid increase in stress triggered the detected earthquake swarm, the lag between peak induced stress and the onset of RTS is much shorter.

(3) Implications to seismic risk: The detection of this particular RTS event has implications to seismic hazard analysis around the Victoria Reservoir. Recent 3-D finite element modelling suggests that the Victoria Dam can withstand peak ground accelerations up to about 0.2–0.3 g with minimal damage^46^. While that level of shaking intensity is almost certainly expected in the near-field of moderate-to-large events (M_W_ ≥ 6), some smaller (M_L_ ~ 4–5; M_L_ ≈ 1.5 M_W_) shallow intraplate events are also capable of producing comparable peak ground accelerations in the near-field^47,48^, making them a non-negligible source of seismic risk. Thus, it is necessary to understand how water level changes of the Victoria Reservoir may drive RTS decades after initial impoundment^5,6,49^. This has become even more important in light of the five felt earthquakes proximal to the Victoria Reservoir within a short time period. Although the recorded peak ground accelerations (PGA) are ~ 0.001g at VILB and ~ 0.0002g at PALK for the largest earthquake (M_W_ = 2.9) that occurred on 29 August 2020 are well below the predicted damaging PGA threshold, the possibility of future moderate-sized events driven by RTS exceeding that threshold in the near-field cannot be ruled out. On the other hand, the risk to the Victoria Dam from repeated shaking of small events is not determined even if PGAs from smaller events do not exceed the damage threshold. If the strength of faults around the Victoria Reservoir has degenerated due to water-rock chemical interactions whose effect can be amplified by mechanical grinding as proposed for the Koyna fault zone^49^, carefully managing reservoir water level fluctuations become imperative to mitigate future risks, which warrants establishing an advance seismic monitoring program.

## Conclusions

We report here the first evidence of RTS in Sri Lanka that occurred in the vicinity of the Victoria Reservoir. The detected triggered seismic swarm that occurred between August and December of 2020 exhibits physical and spatio-temporal characteristics that are consistent with typical RTS episodes. Our predictions based on poroelastic theory, together with probabilistic fault plane solutions, demonstrate that steeply-dipping fault planes oriented in an E–W direction were brought to failure with the decisive action of pore water pressure associated with a major reservoir water level fluctuation of ~ 40 m. Brittle deformational structures parallel to the steeply-dipping E–W oriented Randenigala Fault are candidate causal faults whose weakening process may have been promoted by porewater-rock chemical interactions and mechanically weaker phyllosilicate-rich subparallel bedrock foliations. This study, conducted by optimally exploiting scarce seismic monitoring resources and efficient low-cost computational workflows, can be emulated in other developing countries to perform seismic monitoring and RTS analysis.

## Methods

### Template matched cross-correlation detection

Cross-correlation detection is a powerful seismological tool used to detect repeating earthquakes by scanning continuous three-component seismic waveforms with known template events^50^. In this study, we employed the cross-correlation detector in ObsPy Python package^51^ to scan continuous waveform data recorded on the PALK seismic station (Figs. 1, 2a). In the first instance, we used three-component waveforms of the five felt earthquakes recorded at PALK as templates (Supplementary Fig. S3) and scanned the continuous data between 01 August 2020 and 31 December 2020, following which we detected 11 new events with distinct P- and S-wave arrivals (Supplementary Fig. S4). Combining the templates of initial five felt events with waveforms of the newly detected 11 events, we constructed a new database with a total of 16 event templates, using which continuous seismic waveform archive of the PALK station was scanned from 01 January 2012 through 01 June 2021 (9.5 years). With this procedure, an additional 12 events were detected, bringing the total number of earthquakes in our catalogue to 28 (Supplementary Table S1). All events were detected between 14 February 2020 and 26 May 2021 and no events were detected outside this time window (Supplementary Table S1). Note that we have high confidence in our detections as we have used a waveform similarity cut-off of 0.8, which also means that the total number of events we have presented here is a minimum number as we may have missed events with a waveform similarity less than 0.8. However, we prioritised quality of detections over volume in this instance.

### Earthquake centroid determination

The Sri Lankan seismograph network currently has four permanent seismographs: PALK (a 90 m deep borehole station with minimal ambient noise) in Pallekale, Kandy; MALK in Mihintale, Anuradhapura; HALK in Hakmana, Matara; and BULK in Buddangala, Batticaloa (Fig. 1a). The meridional network configuration with gap angles greater than 120°, relatively large inter-station spacing (> 100 km), and restricted access to seismic waveform data from BULK (operated by the Geological Survey and Mines Bureau; GSMB) are not favourable for locating smaller earthquakes around the Victoria Reservoir using conventional travel time inversion methods. The felt earthquakes initially located imprecisely by the GSMB based on felt intensities^52^ were within 4–5 km of the PALK station with the next nearest broadband station located > 100 km away. This lack of a dense seismic network, close proximity of events to PALK, and the high-quality of waveforms recorded by PALK motivated us to develop a new single station travel-time back-projection location method.

In this method, we first estimated the station-to-source back-azimuth and the incidence angle of first arrival P-wave using the three-component amplitude ratio method^53^ applied to each detection, where we computed pairs of back-azimuth and incidence angles for each incremental time sample of the first arrival P-waveform. The first arrival P-waveform window was first manually picked, which was then refined using an objective automatic waveform envelope-tracking procedure to isolate the part of the wave train that carries a minimum of 70% energy relative to peak energy (Supplementary Fig. S5). We then back-projected travel-times of P- and S-waves for each pair of these angles assuming half-space P-wave velocity models having 5 km/s and 7.5 km/s and Vp/Vs = 1.74. Using this procedure, a candidate set of centroids were isolated by minimising S-P differential travel-times for each event detection. We then obtained the centroid of an event and its uncertainty by, respectively, averaging the coordinates of the candidate set of centroids and computing the standard deviation of them (Supplementary Fig. S6). The procedure we adopted for locating earthquakes, therefore, accounts for measurement and subsurface velocity model uncertainty.

We checked the reliability of our single-station location method using synthetic seismograms. Synthetic seismograms were computed using a frequency-wavenumber double integration method^54^ and a half-space velocity model (Supplementary Table S3) at an array of 1800 hypothetical stations located around a hypothetical source (Supplementary Table S4) with source-receiver azimuths varying from 0° to 360° in increments in 2° and source-receiver distances ranging from 2 to 20 km in increments of 2 km (Supplementary Fig. S7). Applying our single station method to each of these synthetic seismograms with 5% added random noise, we located the hypothetical event as we would with data of PALK (Supplementary Fig. S7). Based on these locations, we estimate mean uncertainty in the lateral location and depth of 2.57 km and 1.59 km respectively. We note that the location uncertainties are higher when the source-receiver azimuth falls within ± 10° of the strike of the nodal planes (Supplementary Fig. S8) and the source-receiver distance is greater than about 10 km. If these stations are removed, we obtain 0.96 km and 0.41 km as the lateral and depth uncertianties, respectively. This synthetic test establishes the optimal operational conditions of our single-station location method and demonstrates that our single-station travel-time back-projection method can be applied reliably to PALK where the back-azimuth is well away from the nodal planes and the epicentral distance is < 10 km.

We also checked the reliability of our single station back-projected locations by comparing them with locations obtained from a conventional travel-time inversion method^55^, and an average velocity model^56^ when travel-time data from a minimum of three stations were available for a given event. For this, we sought data from open access PALK, MALK, and HALK stations (Fig. 1a) as well as dam monitoring strong motion accelerometers installed at Victoria (VILB) and Kotmale (KOLB) with restricted access (Fig. 1c). Note that only the largest two events (29 August 2020; public id = PH20200829s54146 and 18 November 2020; public id = PH20201118s14254) were recorded at distant MALK and HALK stations (Fig. 1a, Supplementary Table S2), and usable data from dam monitoring accelerometers were available only for these same two events. This meant that we could only use two relatively large earthquakes in the conventional travel-time inversion. Nonetheless, we believe that these inversions were adequate to verify the precision of our single-station back-projection method (Supplementary Table S2). Following this comparison, we determined that, for the 29 August 2020 event with travel-time measurements from azimuthally distributed 4 stations, the epicentral location and depth estimates based on our single-station method deviated by less than 1 km from those estimated from conventional travel-time inversion. On the other hand, the epicentral location deviated by ~ 5 km for the 18 November 2020 event that only had three travel-time measurements from stations meridionally located with a gap angle > 120˚. This result is not a reflection of the uncertainty of our single-station method, but the effect of errors produced by conventional travel-time inversion when data are sparse and station locations are suboptimal.

### Focal mechanism determination

First motion polarities of the larger earthquakes on 29 August (public id = PH20200829s54146) and 18 November 2020 (public id = PH20201118s14254) were inverted for focal mechanism solutions using a probabilistic method^59^ (Supplementary Table S2), where solution probabilities are 79% and 74% respectively. The accelerograms were integrated into displacement traces before the inversion. The uncertainty of our solutions is ± 45° and is largely due to the limited number of polarities (at most 4) we have per event. None of the other events met the minimum criterion of having three polarity measurements, precluding the inversion of probabilistic focal mechanisms.

### Earthquake magnitudes

In the absence of an operational ground motion attenuation equation applicable to Sri Lanka, we used a ground motion prediction equation^18^ derived from a sparse dataset to estimate the moment magnitude (M_W_) of detected earthquakes. The M_W_ was estimated for the dominant frequency range between 8 and 20 Hz of the peak ground acceleration (PGA) at PALK. The uncertainty in the predicted magnitudes is expected to be several tenths of a decimal point. We are also reporting the maximum displacement and the hypocentral distance (slant distance) for each earthquake (Supplementary Table S1) so that more reliable magnitudes can be computed once an operational ground motion attenuation equation is established for the Island. If one uses the standard scaling of M_L_ = 1.5 M_W_^57^ to convert Mw to local magnitude (M_L_), the magnitude range of our earthquakes are 0.6 ≤ M_L_ ≤ 4.3.

### Fault stability due to reservoir level changes

Fault stability (S) as defined by the Coulomb-Mohr frictional failure criterion^58,59^ is given in Eq. (1).

1 $$S_{P} = \tau - \mu \mathop \sigma \limits^{^{\prime}} = \tau - \mu \left( {\sigma - P} \right) = \tau - \mu \sigma + \mu P = S + \mu P$$

Here, τ is shear stress, σ is normal stress, *P* is pore pressure and μ is coefficient of friction. We adopt the Green’s function based stress diffusion solution in the porous elastic half-space^37,60^ to model the evolution of stress and pore pressure beneath the Victoria Reservoir. The components of elastic stresses due to the reservoir load are resolved on fault planes determined from our probabilistic focal mechanism inversion to get shear stress and normal stress. For calculating reservoir induced pore pressure both the drained and undrained components are considered. Compressive normal stress is considered positive and shear stress is resolved in the slip direction derived from the earthquake focal mechanism. Positive τ and negative σ promote shear failure due to reservoir impoundment while increase in pore pressure always encourages failure. Thus, failure along the fault is encouraged if change in fault stability is positive. We computed fault stability using two sets of parameter values for hydraulic diffusivity, C (0.1 and 1.0 m^2^/s) and coefficient of friction, μ (0.4 and 0.6) (Figs. 3, 4 and Supplementary Fig. S2). The selected hydraulic diffusivity values are representative of those inferred for crustal depths less than 3 km^60^ around which the detected earthquake swarm is clustered. On the other hand, standard values for friction coefficient are used in our modelling^28^.

## Supplementary Information

Supplementary Information.

## Data Availability

The broadband seismic data used in the study are available from the Incorporated Research Institutions for Seismology (IRIS) (https://doi.org/10.7914/SN/II). Strong-motion accelerometer data and reservoir level data can be obtained from the Mahaweli Authority of Sri Lanka but are not shared with an open-access license. Single station centroid location, and cross-correlation template matching code is hosted on GitHub (https://github.com/pasansherath/sshypo).
