# Supplementary material for: A reservoir induced earthquake swarm in the Central Highlands of Sri Lanka
Source: Sci Rep. 2022 Oct 29;12:18251. doi: 10.1038/s41598-022-22791-z (PMC9617912; doi:10.1038/s41598-022-22791-z)
Supplement: Supplementary file 1 — Supplementary Information. [file 41598_2022_22791_MOESM1_ESM.pdf]

A Reservoir Induced Earthquake Swarm in the Central Highlands of Sri Lanka

Pasan Herath[1,#], Januka Attanayake[2,*], Kalpna Gahalaut[3]

[1]Institute of Geophysics, Victoria University of Wellington, Wellington, New Zealand.

[2]School of Geography, Earth and Atmospheric Sciences, University of Melbourne, Australia.

[3]CSIR-National Geophysical Research Institute, Hyderabad, Telangana, India

[*]januka.attanayake@unimelb.edu.au

#Now at: Department of Earth and Environmental Sciences, University of Ottawa, Ottawa, Canada.

Supplementary Information

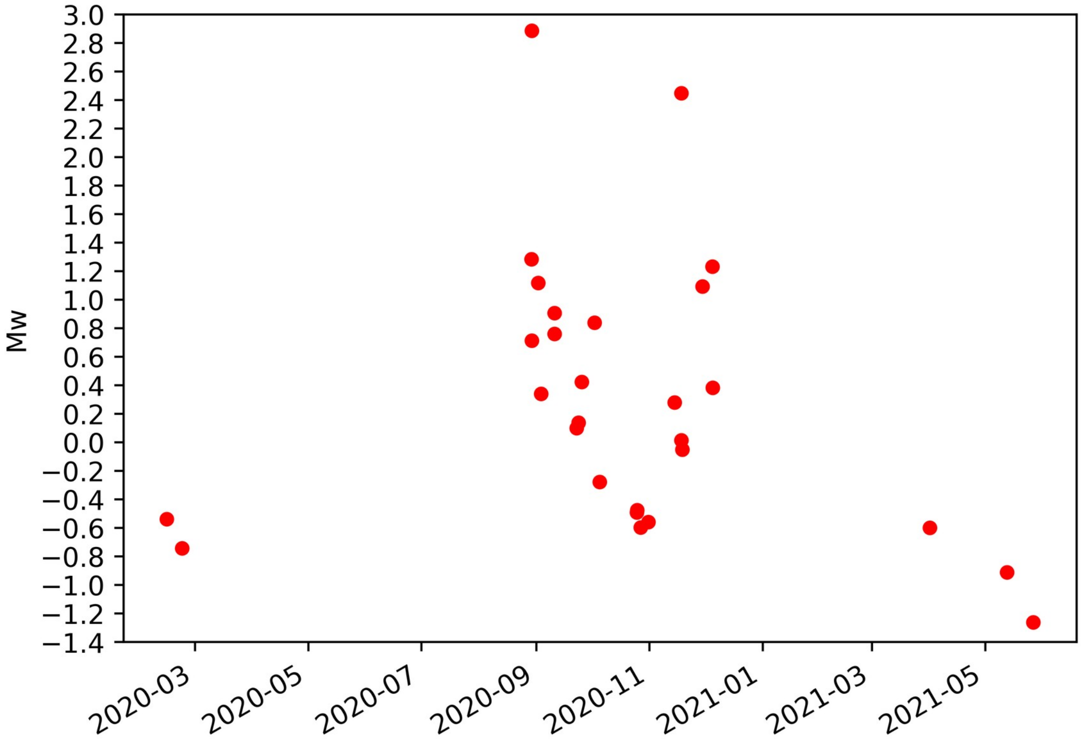

Supplementary Figure S1. Temporal distribution of moment magnitudes (M_W) of the earthquake swarm. Figure compiled using Python Matplotlib[62].

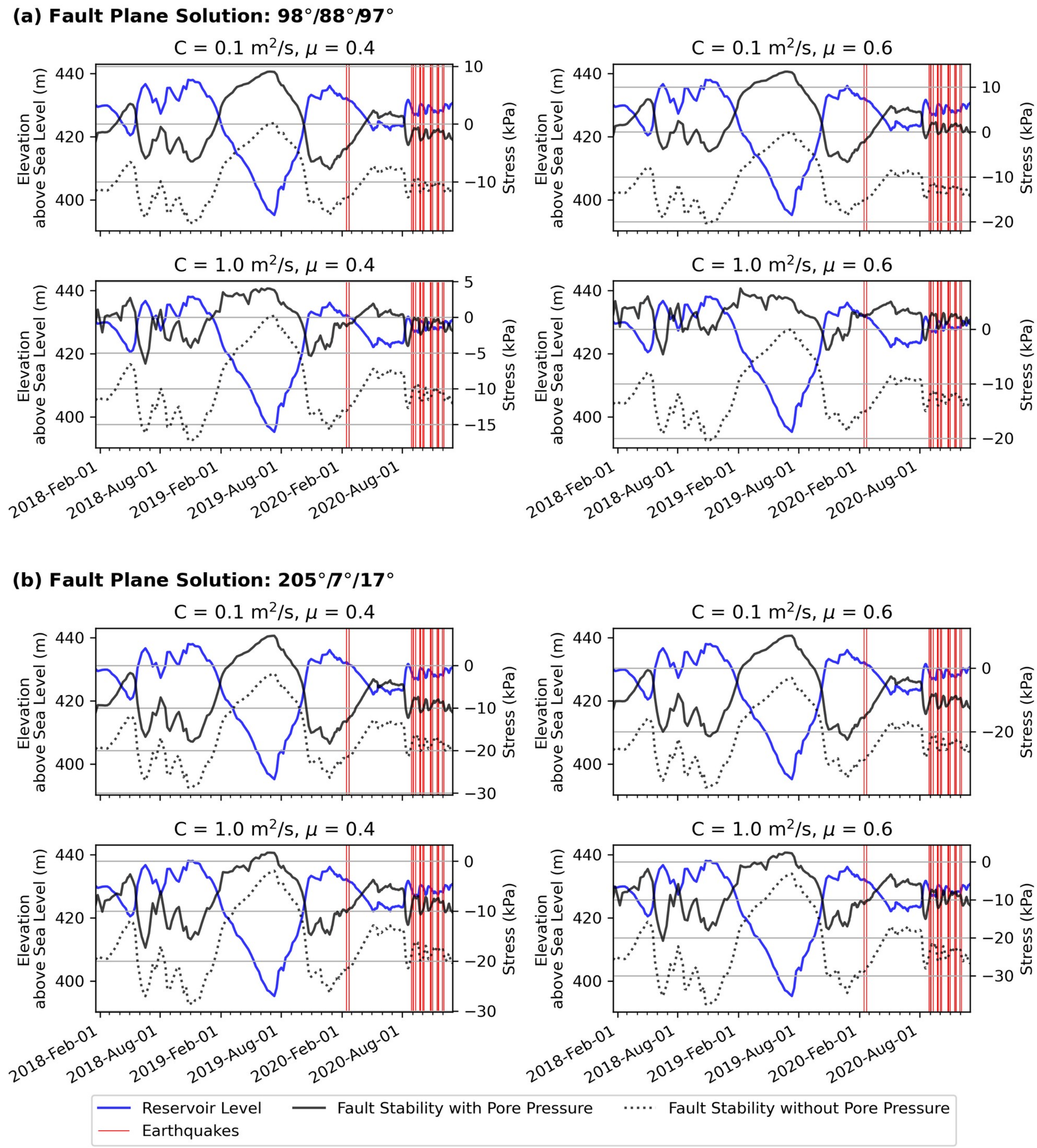

Supplementary Figure S2. Stability of fault plane solutions for the main earthquake on 18 November 2020 in dry (without pore pressure) and wet (with pore pressure) conditions. Failure is encouraged if fault stability is positive. The solutions are for longitude = 80.73842° and latitude = 7.26497 (epicenter of the 29 August 2020 M_W 2.9 event) and depth 2 km. Figure compiled using Python Matplotlib[62].

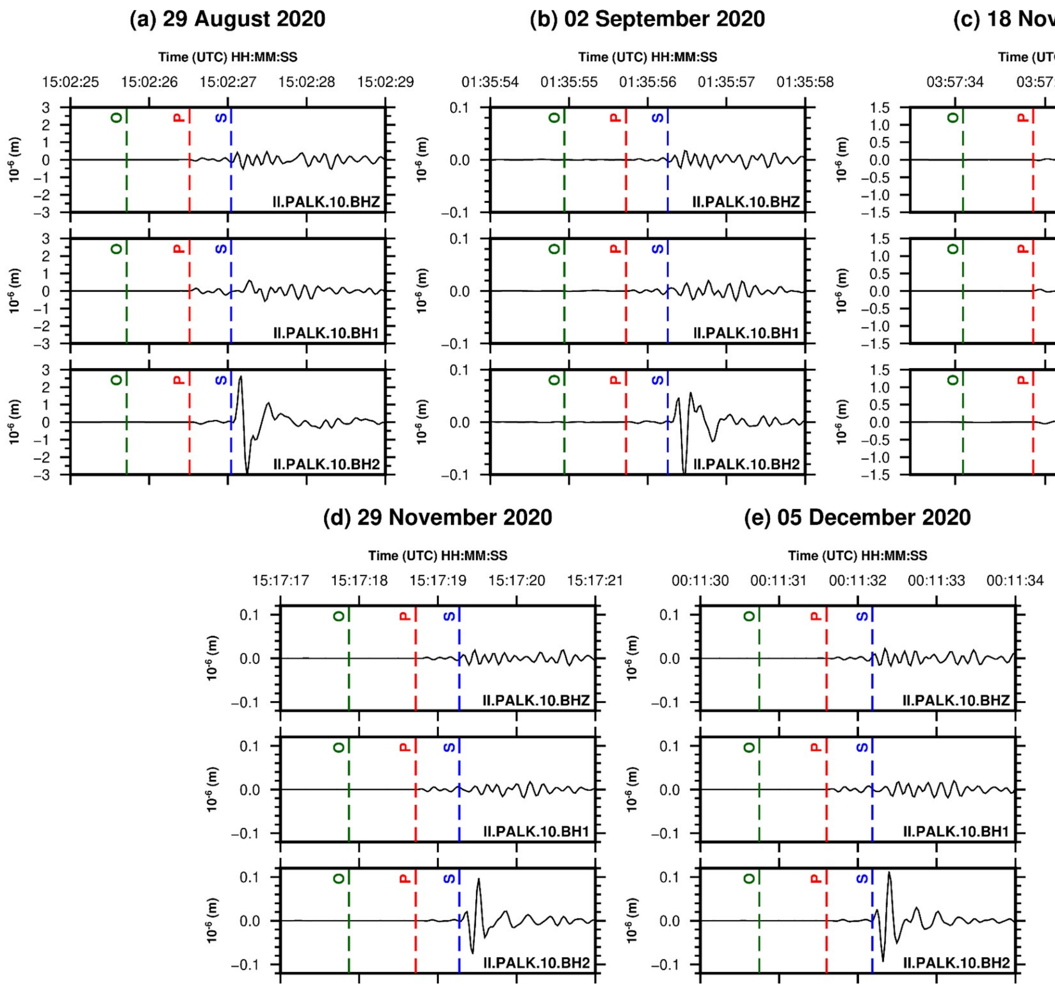

Supplementary Figure S3. (a)-(e) Three component displacement seismograms with the origin (O), P-wave arrival (P) and S-wave arrival (S) times at PALK broadband seismograph of the five felt earthquakes. Figure compiled using Generic Mapping Tools[61].

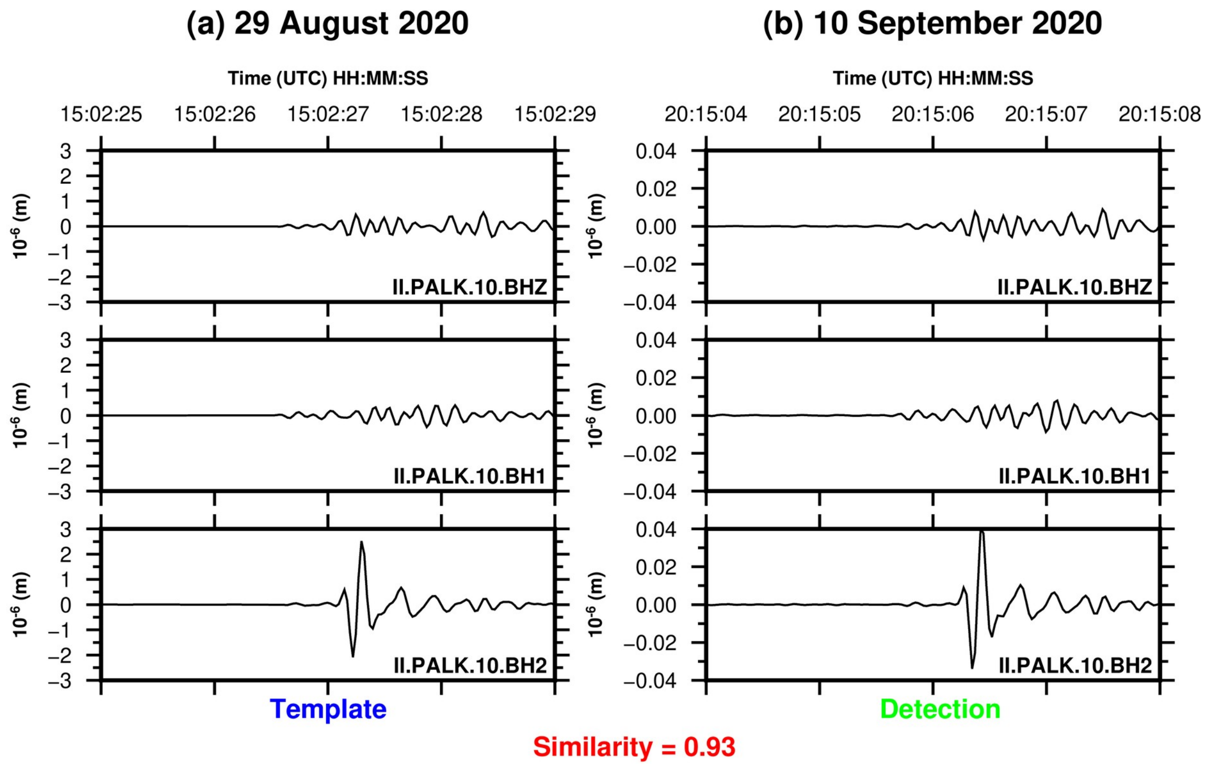

Supplementary Figure S4. Displacement waveforms at PALK of (a) main felt earthquake on 29 August 2020 (template), and (b) a detected earthquake from cross-correlating the template event with the continuous waveforms. Figure compiled using Generic Mapping Tools[61].

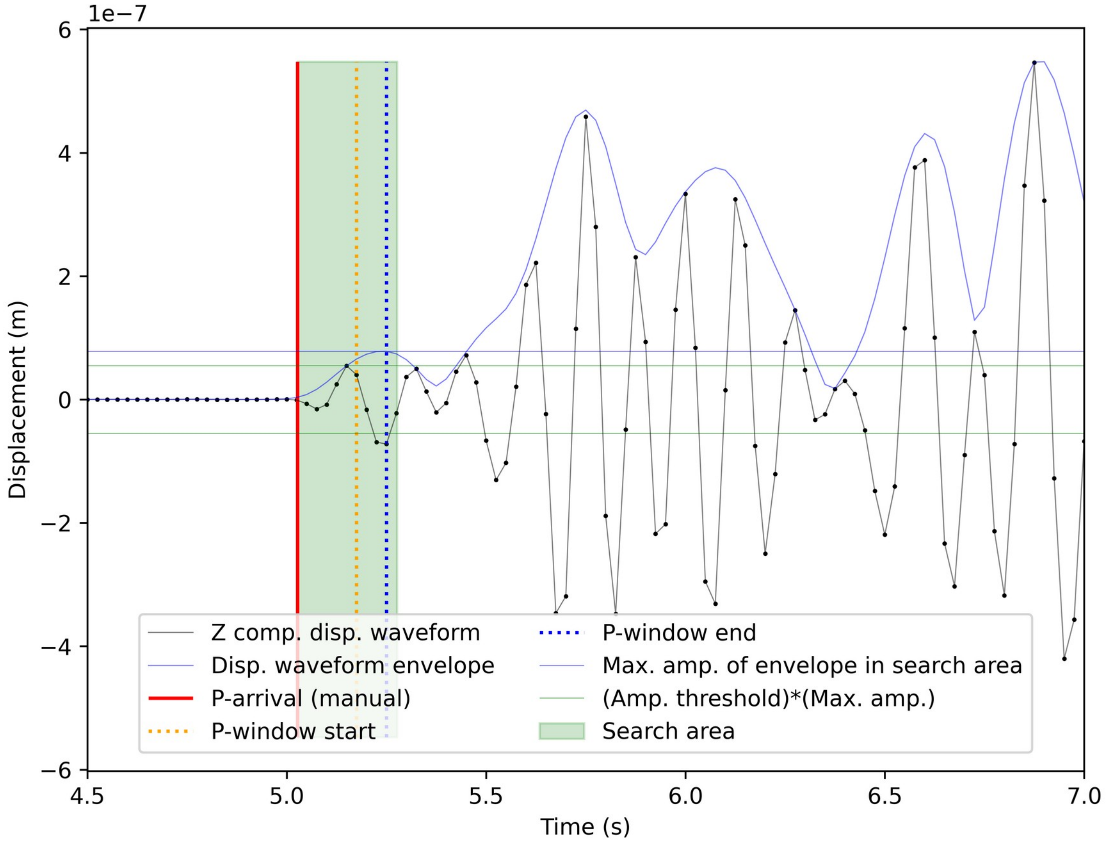

Supplementary Figure S5. Fine-tuning the P-waveform using a threshold of 70% of the peak amplitude of the manually picked P-waveform. Vertical dashed lines indicate the fine-tuned P-waveform window. Figure compiled using Python Matplotlib[62].

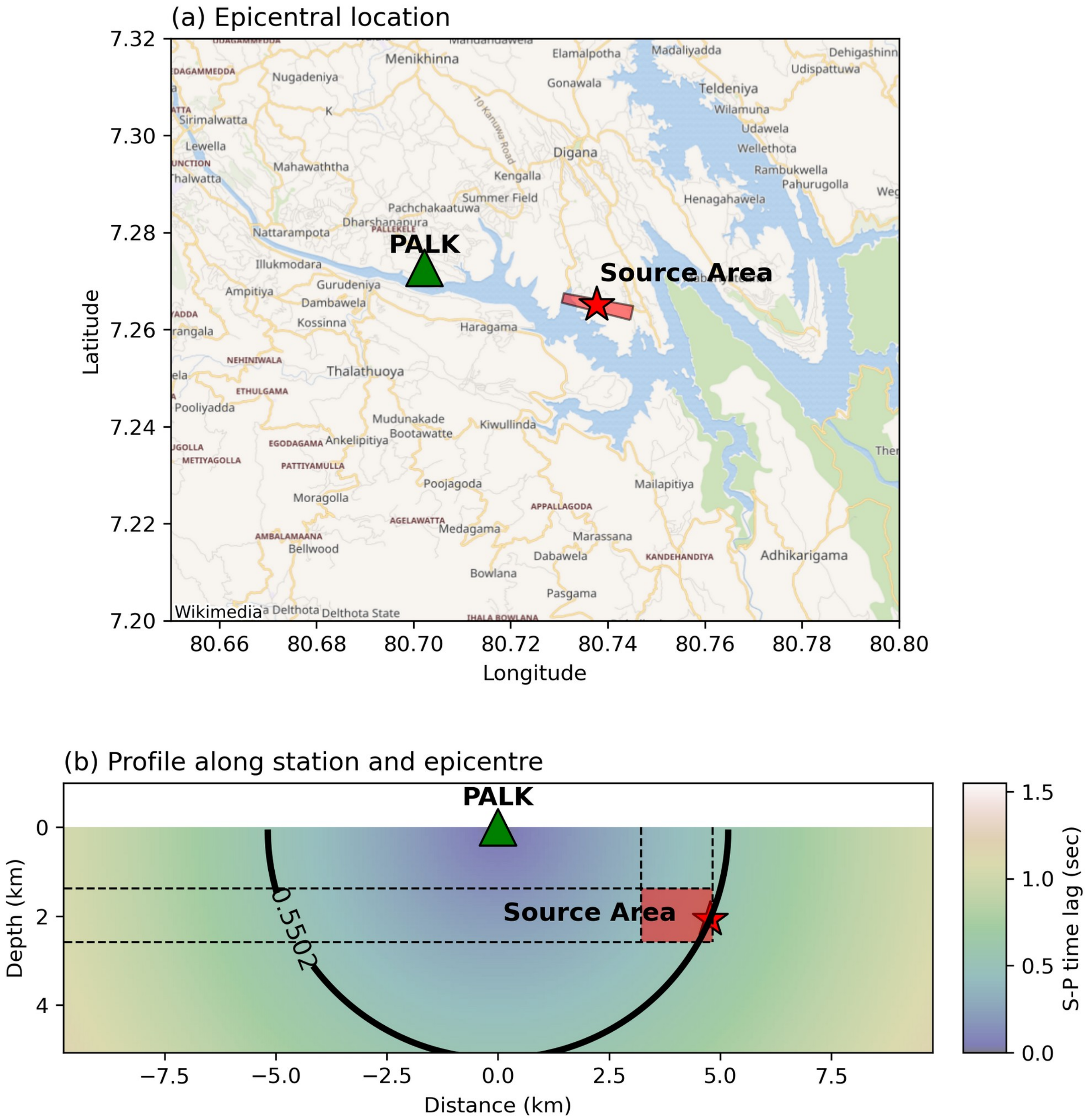

Supplementary Figure S6. Single station hypocentre location of the main earthquake on 29 August 2020. (a) Epicentral location. Red shaded region is the source area based on backprojecting the backazimuths at PALK. Red star denotes the mean epicentral location within the source area. Basemap credit: OpenStreetMap distributed under Open Database Licence (Odbl). (b) Profile along the station and mean epicentre in (a) showing the depth of the source area. Red star indicates the mean depth when Vp=7.5 km/s. Black contour represents the time lag between P- and S-wave arrivals (in sec) for Vp=7.5 km/s. Figure compiled using Python Matplotlib[62].

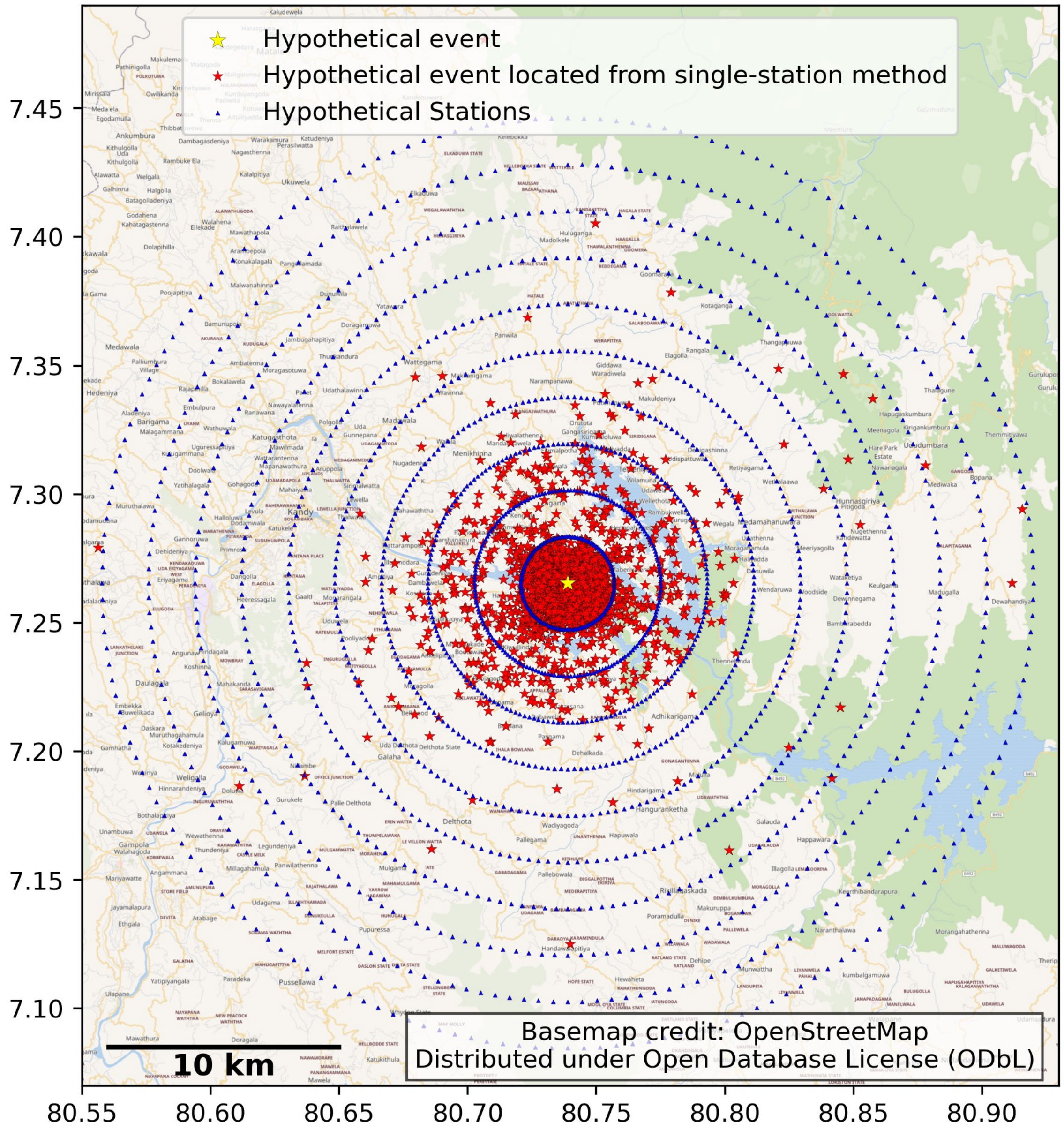

Supplementary Figure S7. Locations of the hypothetical event (yellow star) and the hypothetical stations (blue) around it. Epicentral locations of the hypothetical event located using the synthetic waveforms at the hypothetical stations with the single-station backprojection method are shown by red stars. Basemap credit: OpenStreetMap distributed under Open Database Licence (Odbl). Figure compiled using Python Matplotlib[62].

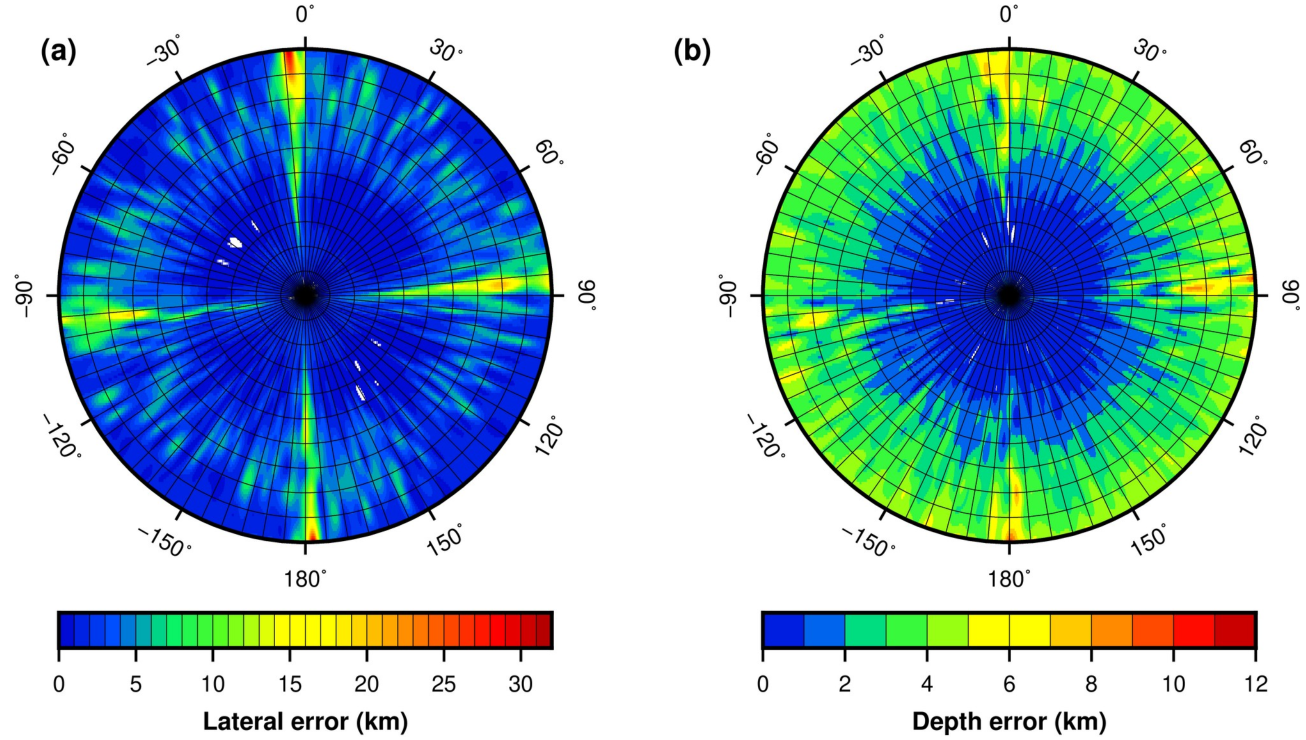

Supplementary Figure S8. (a) Lateral and (b) depth errors in the hypocenter determination of the hypothetical earthquake (Supplementary Table 3) at the hypothetical seismographs (Supplementary Figure 6) using the single station backprojection method. Circles in the polar plots begin at 2 km and end at 20 km with a 2 km interval. Figure compiled using Generic Mapping Tools[61].

Supplementary Table 1. Earthquake catalogue of the study area. Hypocentre estimates are from the single station method. * indicate a felt event.

Public ID	Hypocenter			Mw	Origin time (UTC)	Hypocentral distance (km)	Maximum Displacement (mm)
	Mean Lon.	Mean Lat.	Mean Depth (km)				
PH20200214s69444	80.7235	7.2198	4.67	-0.5	2020-02-14T19:17:22.726968Z	6.37	0.0253
PH20200223s02594	80.7266	7.2491	3.25	-0.7	2020-02-23T00:43:13.499943Z	3.80	0.0146
PH20200829s31696	80.7310	7.2537	1.88	1.3	2020-08-29T08:48:15.960079Z	3.81	0.6360
PH20200829s47972	80.7296	7.2515	1.85	0.7	2020-08-29T13:19:32.185079Z	3.83	0.1680
PH20200829s54146*	80.7338	7.2568	2.10	2.9	2020-08-29T15:02:25.901295Z	3.93	10.8766
PH20200902s05755*	80.7244	7.2489	1.79	1.1	2020-09-02T01:35:55.243862Z	3.64	0.3912
PH20200903s51694	80.7308	7.2424	2.44	0.3	2020-09-03T14:21:33.241160Z	4.63	0.0457
PH20200910s72905	80.7364	7.2567	2.08	0.8	2020-09-10T20:15:04.992511Z	4.17	0.1820
PH20200910s75105	80.7324	7.2516	1.75	0.9	2020-09-10T20:51:45.126295Z	4.09	0.2463
PH20200922s64907	80.7151	7.2442	2.47	0.1	2020-09-22T18:01:47.035079Z	3.47	0.0464
PH20200923s73179	80.7330	7.2588	2.03	0.1	2020-09-23T20:19:38.710079Z	3.74	0.0469
PH20200925s40745	80.7214	7.2366	2.63	0.4	2020-09-25T11:19:04.216160Z	4.56	0.0816
PH20201002s37014	80.7339	7.2604	2.70	0.8	2020-10-02T10:16:53.967509Z	3.79	0.1938
PH20201005s17689	80.7258	7.2413	4.47	-0.3	2020-10-05T04:54:48.147242Z	4.35	0.0232
PH20201025s25210	80.7307	7.2556	2.79	-0.5	2020-10-25T07:00:09.567511Z	3.70	0.0163
PH20201025s30930	80.7299	7.2566	1.52	-0.5	2020-10-25T08:35:29.977646Z	3.54	0.0198
PH20201027s24910	80.7142	7.2526	3.07	-0.6	2020-10-27T06:55:09.568862Z	2.63	0.0145
PH20201031s30745	80.7248	7.2528	3.53	-0.6	2020-10-31T08:32:25.033728Z	3.34	0.0136
PH20201114s50033	80.7334	7.2563	2.55	0.3	2020-11-14T13:53:52.842511Z	3.90	0.0908
PH20201118s14254*	80.7298	7.2525	1.89	2.4	2020-11-18T03:57:34.285079Z	3.81	5.7897
PH20201118s14413	80.7189	7.2550	2.75	0.0	2020-11-18T04:00:13.302646Z	2.70	0.0428
PH20201118s65469	80.7257	7.2553	3.62	-0.1	2020-11-18T18:11:08.583727Z	3.25	0.0322
PH20201129s55038*	80.7334	7.2584	1.90	1.1	2020-11-29T15:17:18.160077Z	3.81	0.4257
PH20201205s00691*	80.7330	7.2575	1.88	1.2	2020-12-05T00:11:31.035079Z	3.81	0.4494
PH20201205s09010	80.7304	7.2642	2.72	0.4	2020-12-05T02:30:09.760079Z	3.26	0.0856
PH20210401s25065	80.7179	7.2377	4.31	-0.6	2021-04-01T06:57:44.631024Z	4.26	0.0364
PH20210512s73116	80.7126	7.2114	3.44	-0.9	2021-05-12T20:18:34.785754Z	6.89	0.0148
PH20210526s81139	80.7213	7.2481	3.64	-1.3	2021-05-26T22:32:18.649943Z	3.49	0.0096

Supplementary Table 2. Hypocentre and focal mechanisms of felt earthquakes from travel-time and probabilistic inversion.

Public ID	Travel-time Inversion (Multiple Stations)				Difference from Single Station Hypocentre Estimate		Fault Plane Solutions (Strike/Dip/ Rake)
	Stations used	Lon.	Lat.	Depth (km)	Epicentre (km)	Depth (km)	
PH20200829s54146	PALK<br>MALK<br>VILB<br>KOLB	80.7407	7.2599	2.53	0.84	0.36	128°/18°/-28°<br>265°/77°/-10°
PH20201118s14254	PALK<br>MALK<br>HALK	80.6888	7.2617	1.96	4.64	0.07	205°/7°/17°<br>98°/88°/97°

Supplementary Table 3. Parameters of the halfspace seismic velocity model used to compute synthetic seismograms at the stations given in Supplementary Table 1 for the hypothetical source given in Supplementary Table 2.

Layer thickness (km)	S-wave velocity (km/s)	V_P/V_S	Density (g/cm^3)	Q_S	Q_P
60	3.468	1.73	2.60	600	1300

Supplementary Table 4. Parameters of the hypothetical source used to compute synthetic seismograms at stations in Supplementary Table 1 using the halfspace velocity model in Supplementary Table 3.

Longitude	Latitude	Depth (km)	Mw	Strike	Dip	Rake
80.73888	7.26542	2	2.0	265	77	-10
